# Sett Use, Density and Breeding Phenology of Badgers in Mediterranean Agro-Sylvo-Pastoral Systems

**DOI:** 10.3390/ani11092663

**Published:** 2021-09-10

**Authors:** Marcelo Silva, Luís Miguel Rosalino, Sandra Alcobia, Margarida Santos-Reis

**Affiliations:** cE3c—Centre for Ecology, Evolution and Environmental Changes, Faculdade de Ciências, Universidade de Lisboa, 1749-016 Lisboa, Portugal; Marcelo_Gomes_Silva12@hotmail.com (M.S.); lmrosalino@fc.ul.pt (L.M.R.); alcobiasandra@gmail.com (S.A.)

**Keywords:** camera-trapping, *Meles meles*, density, social organization, reproduction

## Abstract

**Simple Summary:**

Understanding carnivores social structure variation is pivotal for properly addressing conservation challenges and solutions. The European badgers is a social carnivore for which most of the available information regarding how this species is socially organized derives from central west populations. This article describes the group composition, den use patterns and breeding phenology of a Mediterranean population of badgers. We showed that badger live in low density, in relatively small groups, composed by 2–4 adult animals and ca. 2 cubs, born in winter. These patterns, representing a variation of what was described for other populations, show that badgers take advantage of the landscape context, where human-related resources and mild environmental conditions allow badger to reach higher densities than in many southern populations, and to reproduce earlier than their northern counterparts.

**Abstract:**

Carnivores social organization varies widely, from strongly social to solitary predators. European badgers are facultative social carnivores that also shows a geographical variation in social structure. These patterns derive mainly from central/west European regions, with an under-representation of Mediterranean populations that face different conservation challenges, especially regarding group composition, sett use patterns and breeding phenology. We addressed these traits topics for a population inhabiting a Portuguese agro-silvo-pastoral system. Based on monthly monitoring of 34 setts and continuous camera-trapping surveys of 12, we showed that setts surrounded by diversified vegetation and located in sandy sites are more used, a pattern probably linked to food availability and ease of sett excavation and maintenance, respectively. Badgers followed a general pattern regarding group size (2–4 adults), but showed an intermediate population density (0.49–0.73 badgers/km^2^), with values higher than those estimated for other Mediterranean environments, but lower than for central-western populations. This, together with the breeding (November/January) and cub emergence (1.8 cubs/sett; March/April) periods, indicates an ecological adaptation to the landscape context, where human-related resources and mild environmental conditions allow badger to reach higher densities than in many southern populations, and to reproduce earlier than their northern counterparts.

## 1. Introduction

Carnivores show a wide array of social organizations, ranging from solitary (e.g., wildcats, *Felis silvestris*) to highly social species, such as the meerkat (*Suricata suricatta*) [1]. However, less than 20% are considered social predators [2], and even those show distinct degrees of sociality. While wolves (*Canis lupus*) can form packs with more than 40 animals sharing hunting, territory patrolling and defense, cub rearing, and other activities [3], others, such as European badgers (*Meles meles*), form clans usually with less than seven individuals that share the same refuge and territory and show some intra-clan interactions, but lack the social sophistication showed by highly social species [4]. The way badger social groups are organized and use the communal refuges they build (known as setts) is however highly variable across its distribution range and this variability still poses questions about how the landscape context may shape this species’ social patterns.

Throughout Europe, the size of badger social groups ranges from singe pairs in southern Spain [5] to seven adult individuals in Wytham Woods, UK (although reaching 30 adults/yearling badgers in unusual situations, such as those found in Woodchester Park, UK, in 1989) [6]. Such social structure determines the species density, which peaks in the UK (38 ind./km^2^) and reaches its lowest value in Eastern Europe (e.g., Czech Republic, 0.12 ind./km^2^ [7]. This variation in density is frequently linked to climate variation (e.g., wetter climate favors earthworm’s abundance and, indirectly, badgers), abundance and availability of potential sett sites and/or food, and level of disturbance, such as human population density, road density or hunting pressure [8].

Iberia, corresponding to the species south-western range limit, seems to be a challenging environment for badgers, which occur at low densities (0.13–0.67 ind./km^2^, in Spain, and 0.36–0.48 ind./km^2^ in Portugal) [9,10,11,12]. This population structure seems to be determined mostly by the low availability of food resources [11] or sett sites [13] in Mediterranean environments. However, estimations of badger density are limited to very few regions in Iberia—Doñana National Park (S) and Park of Collserola (NE), in Spain, and Serra de Grândola in Portugal [10,11,12]—not representing the full range of environmental conditions badgers explore in Iberia. Thus, more data are needed to allow a better understanding of how Iberian populations respond to and are influenced by the Iberian landscape context, because this region is expected to suffer drastic environmental changes in the next future due to climate warming [14,15].

Setts play a central role in badger socio-ecology, acting not only as refuge sites (where individuals spend as much as 70% of their time), but also as focal points for reproduction, a nursery, and a social exhibition and interaction arena [4]. For all these reasons, badgers density is often linked to sett density, especially when considering the main setts, i.e., larger setts (>5 entrances) with signs of a regular use throughout the year [16]. However, in low density areas, as Iberia, this relation is weaker, and keeps valid only including also smaller and less frequently used setts (often called, secondary, outliers or subsidiary) [16]. Whatever the type of setts considered, their use pattern is determined by their internal structure, landscape context and disturbance risk [17,18,19].

In the Mediterranean region, human presence has shaped the landscape for millennia, converting natural environments to agriculture land, pasture for livestock or human settlements/infrastructures [20], affecting wildlife ecological patterns. In Iberia, badgers show low sett use rates, with animals frequently changing between setts in consecutive days (ca.50% of the occasions), and the re-use rate greatly declining after five nights [21]. This pattern seems to be linked to anthropic disturbances, such as cattle breeding and removal of forest understory around the setts [22].

Setts are also a pivotal structure in badger reproduction, as birth occurs in underground sett chambers, and cubs stay inside until they can explore the outside environment [4]. While mating can occur throughout the year, most available birth records are concentrated in February, due to delayed implantation, with a litter size of 2–3 cubs (range 1–5) [4]. However, this information originates mainly from UK populations, with scarce data available for Mediterranean populations, where the highly different climate may produce variations to the British pattern. Available data for the Iberian Peninsula indicate that in captive animals mating occurred between February and May, with the birth of 1–3 cubs in February [23], while 3–4 cubs per sett have been reported for a wild population in Portugal [12].

Considering the specific characteristics of Mediterranean landscapes, and the lack of information on the ecology of badgers in this region, our study aimed to assess badger: (1) density; (2) sett use patterns and underlying determinants; and (3) breeding phenology and litter size.

To fulfill these goals, we tested eight hypotheses (Table 1).

## 2. Materials and Methods

### 2.1. Study Area

**Figure 1:**
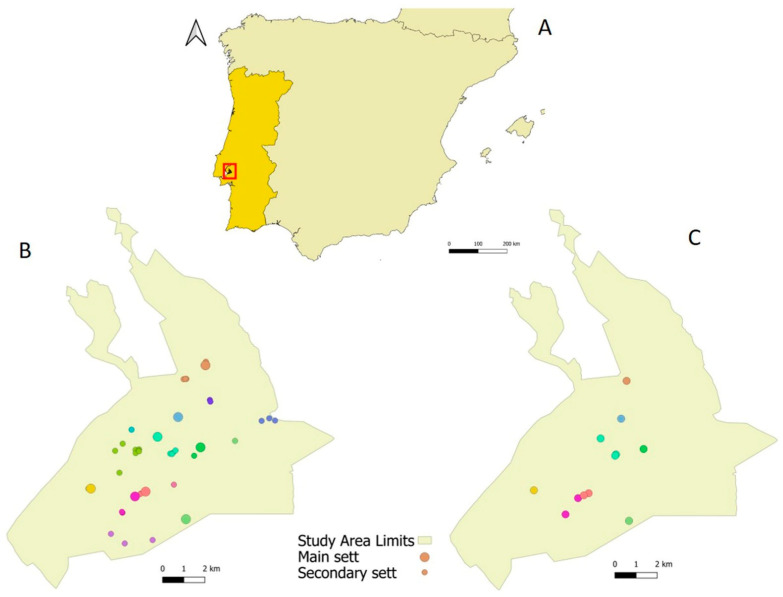
Study area location (**A**), and spatial distribution of detected badger setts (**B**), highlighting those selected for intensive monitoring using camera-traps (**C**). Different colors represent distinct badger social groups, and main setts are represented by bigger circles.

The study was conducted in the “Charneca do Infantado” (38°48′ N 8°49′ W; Figure 1), a 100 km^2^ farmstead managed by Companhia das Lezírias S.A., mainly devoted to the production of cork (cork oak, *Quercus suber*, woodlands—67.50 km^2^), wood (maritime pine, *Pinus pinaster*, plantations—9.71 km^2^; Eucalyptus, *Eucalyptus globulus*, plantations; 4.76 km^2^), pine nuts (stone pine, *Pinus pinea*, plantations—5.08 km^2^), and agricultural goods (e.g., corn, *Zea mays*—2.50 km^2^; rice, *Oryza* spp.—2.40 km^2^; olives, *Olea europaea*—0.70 km^2^). This multiuse agroforestry system also includes other activities, namely seasonal cattle raising, with cattle grazing in the farmstead in Autumn/Winter, and hunting mostly of wild boars (*Sus scrofa*) and partridges (*Alectorys rufa*), for which several artificial feeding stations with cereals and water are available throughout the farmstead [35].

The region has a typical Mediterranean climate, with hot and dry summers and mild but rainy winters. During the study period the mean temperature and precipitation were of 16.8 °C and 361 mm, respectively. “Charneca do Infantado” is located in a plain lowland area, with few relatively deep valleys formed by temporary watercourses. Only one permanent stream crosses the farmstead (“Ribeira de Vale Cobrão”), although several artificial water points and temporary ponds are scattered throughout the areas.

### 2.2. Sett Survey

We first reviewed and compiled all sett records registered in previous studies conducted in the study area (e.g., [22,36]). All these setts (*n* = 33) were revisited to confirm their use by badger and new setts were intensively searched in the study area. The area is divided in cattle grazing plots and, between September 2016 and April 2017, we surveyed 20 that fulfilled the characteristics considered promoters of sett emplacement (vegetation and soil features; see [4]; and avoiding intensive agriculture areas, such as rice and corn fields, water channel and reservoirs, etc.). These plots, totalizing 15 km^2^ (mean area 1.45 km^2^, sd = 1.05, range: 0.05–6.66 km^2^) were surveyed based on several linear and parallel transects, covering the entire cell. The distance between the transects was about 50 meters, since we surveyed a buffer of 25 m in each perpendicular direction. In more closed areas (woodlands, for example), the distance between transects was shortened to ensure that the whole area was surveyed, as the visual detection of setts decreases in dense vegetation areas.

Assuming that main setts are located in the core area of each group territory [24], and considering that badgers home range in a similar Portuguese landscape was 4.46 km^2^ [12], we assumed that all secondary setts located within a radius of 1191 m from a main sett (see Section 2.3) will belong to the same social group. The only exceptions were secondary setts either separated by an insurmountable geographical barrier (e.g., permanent stream) or located within the buffer boundaries of two neighbor main setts (in such situation they were grouped with the closest main sett).

### 2.3. Sett Monitoring

To determine the sett use pattern, all detected setts were visited monthly to determine badger signs of presence, and estimate a sett use index, ranging from 0 (never active) to 7 always active. A sett was considered active if nearby its entrances we found: (1) freshly excavated soil, (2) recently used latrines; (3) fresh nest material (Figure S1; Supplementary Material); and/or (4) high abundance of footprints.

Setts that showed frequent and abundant activity signs (sett index = 6–7), or where reproduction was detected, were considered main setts [12], all the remaining being classified as secondary [24]. The main setts were the target of an intense monitoring program, using a camera trapping approach [37]. We installed 14 Moultrie^®^ m-990i Gen2 (Calera, AL, USA) camera-traps (Figure S2; Supplementary Material), equipped with a 16 Gb memory card, set to take 30s low resolution videos (854–480 pixels) when the heat/movement sensor was activated, with a minimum time interval between consecutive videos of 5s. Cameras were installed 30 cm above the ground, attached to a tree or a wooden stick, facing one or more active sett entrances. In two setts with more than one entrance active, and that could not be simultaneously monitored with a single camera, an additional one was set to ensure the monitoring of all active entrances. Cameras were checked weekly to replace the batteries and memory cards. Sett monitoring occurred between November 2016 and April 2017, and each camera was active for 180 days, for an overall monitoring period of 2520 camera-trapping nights.

### 2.4. Sett Characterization

In order to assess which environmental drivers, were explaining the sett use pattern, we used 21 variables to characterize each sett (Table 2). Variables associated to vegetation composition and structure were assessed, in situ, within the minimum convex polygon that encompassed all the sett entrances, plus a buffer of 2 m. The Simpson diversity index [38] was estimated based on the herbaceous, shrub and bare soil cover in each polygon. Later, we built a Geographic Information System (GIS; QGIS^®^ version 2.8.3—Wien, Beaverton, OR, USA [39]), that encompassed remote sensing information regarding the: type of soil (data provided by the Companhia das Lezíria, S.A, Samora Correia, Portugal); land use [38]; location of setts, game feeding stations and watering pivots, paved and unpaved roads and water points (to allow the estimation of the distance of each sett from these landmarks; data provided by the Companhia das Lezíria, S.A.); grazing pressure per plot (data provided by the Companhia das Lezíria, S.A.); and wild boar occurrence determined in a previous study [40].

### 2.5. Group Size and Breeding Phenology

Each video was carefully viewed and we registered the number of individuals identified, the age class (i.e., adult, cub; based on animal’s size and survey month [4], the interaction between the animal and the sett (i.e., entering or leaving the sett, excavating or handling nest material), as well other behaviors or social interactions (e.g., antagonistic or breeding behaviors). To estimate the number of individuals per social group we used the highest number of badgers recorded simultaneously. However, this estimation was only considered valid if that number of individuals was observed in, at least 1% of the total recorded videos of each sett. This criterion was defined to reduce the risk of counting, as members of the monitored group, individuals from other social groups that may be visiting the main sett of another group. This conservative approach considers that all badgers detected simultaneously belong to the same social group.

Regarding reproduction, we considered that a mating event occurred when we detected in the video a sexual interaction between two adult badgers. When in subsequent videos the mating event still occurred, we registered the entire sequence as a single mating event. Mating events less than 2 minutes long were considered short-duration mating or copulation events, and those that occurred for more than 2 minutes (typically 12 minutes or more; see results) were registered as long-duration mating events [34]. Long mating events are expected to increase breeding success [41].

### 2.6. Data Analysis

#### 2.6.1. Drivers of Sett Use

In the modelling procedure applied for assessing the environmental drivers of sett use by badgers (hypotheses H2–H6), the number of monthly visits positive for signs of activity (ranging from 0 to 7) was included as dependent variable (Table 1 and Table 2).

Due to the high number of variables associate to H2 we conducted a Principal Component Analysis (PCA) [42], using the variables tree, shrub, herbaceous and bare soil cover and shrub height (Table 2); principal components that cumulatively explained >80% of the information of the original variables, were used as candidate drivers in the subsequent modelling procedure (see 3.1).

Spatial autocorrelation of data was assessed using the Moran I Index [43], and we evaluated multicollinearity between all candidate drivers (except those used in the PCA) using the Spearman correlation coefficient, ρ [42]. When two variables were highly correlated (ρ > 0.70), we excluded the one less correlated with the dependent variable [44].

For each hypothesis, we run Generalized Linear Models (GLM) [42], using a Poisson distribution and a “log” link function, corresponding to all combination of the variables associated to the hypothesis (Table 2). Resulting models were ranked according to the Akaike Information Criterion, with correction for small sample size (AICc) [45]; those that showed a ΔAICc < 2 (i.e., the difference between the AICc of a model and the lowest AICc value in the model set [46]) were considered the best models in explaining sett use variation. If more than one model fulfilled this criterion, we used a model averaging procedure [47] to assess variables average coefficient and the 95% confident interval (95% CI). Variables with a 95% CI that did not include the 0 (i.e., we can determine more precisely if its effect is positive or negative), and that showed a higher relative importance, were considered more influential [48]. Relative importance was estimated as the sum of the Akaike weight (w; probability of a model being the best model) [45], of all the models that included the variable of interest.

The variables that, for each hypothesis, showed a significant (*p* < 0.05) or almost significant (*p* < 0.1) influence on sett use pattern, were included in overall models (combined hypothesis), that postulated that the pattern of sett use is determined by a combination of drivers linked to distinct ecological/environmental processes (vegetation, soil, food, disturbance or competition).

Finally, we compared the AICc of the best model of each hypothesis and considered the hypothesis with more support as that showing the lowest AICc value. The goodness of fit of the best overall model was assessed by estimating the R^2^, which identifies the proportion of variability of the original data explained by the model [47]. Finally, we also tested the autocorrelation of models residuals [46].

#### 2.6.2. Badger Density Estimation

We used the maximum number of different animals detected in each main sett (see above) to estimate the mean number of animals per social group for the entire study area. We then estimated badger density as:

$$\frac{\mathrm{Mean}\;\mathrm{number}\;\mathrm{of}\;\mathrm{adult}\;\mathrm{badgers}\;\mathrm{per}\;\mathrm{main}\;\mathrm{sett}\;\mathrm{estimated}\;\mathrm{for}\;\mathrm{the}\;\mathrm{study}\;\mathrm{area}\;\times \;\mathrm{Estimated}\;\mathrm{number}\;\mathrm{of}\;\mathrm{social}\;\mathrm{groups}\;}{\mathrm{Surveyed}\;\mathrm{area}\;\left(43{\;\mathrm{km}}^{2}\right)}$$

We assumed that the studied badger population followed the behavioral pattern described for other areas of its distribution range, with each social group having one main sett, used all year round and where reproduction occurs [49].

## 3. Results

Of a total of 33 setts identified in the area in the frame of previous studies, only 22 still occurred (i.e., were not destroyed). Twenty-three new setts were added as a result of the field work, totalizing 45 setts for monitoring purposes (Figure 1B), resulting in a sett density of 1.05 setts/km^2^.

### 3.1. Drivers of Sett Use Pattern

From the 45 setts we detected in the study area, we only used 34 in our modeling procedure, since 11 were only detected close to the end of the study not providing robust data regarding their use by badgers. No significant spatial autocorrelation was detected (Moran I = −0.053; *p* = 0.822). From all the candidate co-variates we excluded “Grazz_07–17” and “Grazz”, since they were significantly correlated with “Grazz_16–17 (rGrass_07–17–Grazz = 0.77, *p* < 0.001; rGrass_07–17–Grazz_16–17 = 0.77, *p* < 0.001; rGrazz–Grazz_16–17 = 0.79, *p* < 0.001), and the later was more correlated with the dependent variable (rs = 0.258). Furthermore, we also excluded “Dist_piv” as it was significantly correlated to “Dist_ol” (r = 0.97, *p* < 0.001), and the “Dist_ol” was more correlated to the frequency of sett use (rs = −0.237).

The first two compoments of PCA analysis produced for the vegetation associated variables reached a cumulative explained variance of 80.1% and thus were used as candidate variables in models procuced to test Hypothesis 2 (Vegetation drivers). The association between both components and the original variables is presented in Table 3.

We produced a total of 91 models, but only a few were considered the best models (i.e., ΔAICc < 2) for each hypothesis: four for H2, one for H3, two for H4, three for H5 and two for H6 (Table 4). From the variables included in these best models, only “Vegetation diversity”, “Distance to the nearest olive yards”, ”Main soil material”, “Distance to the nearest paved road”, “Distance to the nearest dirt road” and “Grazing pressure in 2016–2017” had a significant influence in the dependent variable (*p* < 0.001) and thus were used as candidate variables for the combined model (Table 4).

Models for the combined hypothesis showed the highest fit (i.e., lower AICc and Overall ΔAICc < 2). Best models included the variables “Main soil material”, “Vegetation diversity”, “Distance to the nearest dirt road” and “Grazing pressure in 2016–2017”. Based on the model averaging procedure we can state that setts that were more used by badgers during the study period were located in sites with a higher diversity of vegetation (i.e., higher Simpson diversity index), higher levels of grazing in the 2016–2017, and softer soils (e.g., silt/clay soils were avoided) respect to poorly used setts (Table 5).

The best model was able to explain ca. 40% of the variance in the frequency of sett use (R^2^ = 0.397), indicating an acceptable goodness of fit [42], and no spatial autocorrelation of residuals was detected (Moran I = −0.09; *p* = 0.55).

### 3.2. Social Groups Size and Density Estimates

We identified 12 setts that showed a higher and more continuous activity patterns (Figure 1C) and were therefore considered main setts. On average main setts included 3.85 ± 3.32 (ranging from 1–7) entrances, 3 ± 2.2 of which were active simultaneously (i.e., with footprints, latrines, revolved soil, bedding material; range 1–6). Secondary setts had only an average of 1.41 ± 1.66 active entrances, ranging from 0 to 4 (often none showed signs of activity), from an overall 2.93 ± 2.80 total entrances (range: 1–9). The index of sett use was higher for main setts (6.14 ± 1.22) than for secondary setts (3.37 ± 2.23).

Overall, we manage to register badgers activities in main setts on average 150 ± 26 night per set, representing 18,966 videos of 30 seconds (i.e., 158 hours of video). In 46% of the videos (*n* = 8654) we detected at least one badger, and in 7% (*n* = 1281) other mammal species: cattle, wild boars, wild rabbit (*Oryctolagus cuniculus*), common genet (*Genetta genetta*), stone marten (*Martes foina*), red fox (*Vulpes vulpes*) and Egyptian mongoose (*Herpestes ichneumon*). In 41% of the monitored nights we detected badgers interacting with the sett, but in 15% they simply passed through the sett area.

Data from the 12 setts were clustered into eight social groups, based on the criteria defined (see methods; Figure 1). The presence of other setts, located far outside any of the buffers defined around the main setts, indicated the presence of, at least, four aditional groups, within the study area, for which we were not able to detect the main sett. Thus, we estimate the presence of 12 social groups of badgers in our study area.

Based on camera-trapping data, group size ranged from two to four adults, with an average of 2.63 ± 0.744 adult badgers. Based on this group size average, the number of social groups identified (*n* = 12) and the overall area surveyd (43 km^2^), we estimated a badger density of 0.73 badgers/km^2^ (range: 0.53–0.94 badgers/km^2^). However, there is a possibility that the mentioned setts located far away from the known main setts might be outliers, and therefore if we exclude those four assumed groups (for which no main sett was detected), these estimates decrease to 0.49 badgers/km^2^ (range: 0.35–0.63 badgers/km^2^).

### 3.3. Breeding Phenology

**Figure 2:**
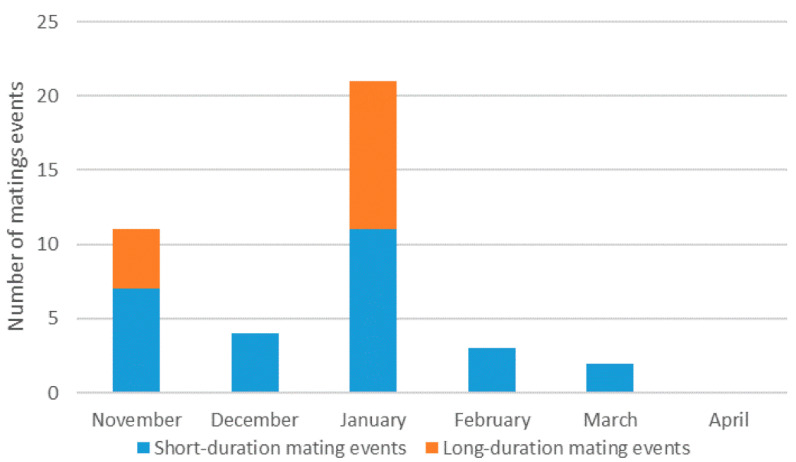
Number of mating events (short and long-term mating events) per months registered in the video-trapping monitoring.

We detected mating behaviours in six of the 12 setts monitored by camera-trapping, for a total of 41 mating events. These mating events were observed throughout the entire monitoring period, with the exception of April (Figure 2). However, long-duration mating events were only detected in November and January (*n* = 21), which were the periods with the highest number of events (Figure 2).

Overall, we registered a higher number of short-duration (*n* = 27) than long-duration (*n* = 14) mating events, the later ranging from 12 minutes to 75 minutes.

Cubs were detected in only five out of the 12 monitored setts (Figure S3; Supplementary Material), being registered in 589 videos (6.8% ± 7.57% of the records where badgers were detected, a percentage that varied from 3.85% to 34.81% in the five setts where cubs were recorded). On average we estimated an average of 1.8 cubs/sett, with two different cubs identified in four main setts and only one in the fifth. Cubs were first detected outside the sett on March 4 in one of the setts, but detection become more frequent after March 15 in most setts. However, in one sett cubs emerged for the first time only in April 4. In one of the setts, but only in two occasions, we detected four cubs simultaneously, although two of them seemed to be older (i.e., they presented a marked bigger body size), which my indicate they were part of a different litter, born in a different period in a nearby sett, which was not monitored.

## 4. Discussion

The European badger shows a high adaptability throughout its wide distribution range highlighted by its wide variation in feeding habits [50], density [7], group size [7], or habitat use patterns and drivers [8]. Our results bring novel information on still poorly known aspects of Mediterranean badger ecology (e.g., drivers of sett use pattern, density, social groups structure and breeding phenology) confirming its ecological flexibility.

### 4.1. Drivers of Sett Use Pattern

The monitored badger population showed a variation in the frequency of sett use, and this heterogeneity in how they utilize the available dens seems to be driven by three different, but complementary drivers, that highlight the importance of regional characteristics: vegetation structure, soil composition and disturbance. Such effect of distinct factors supports our hypothesis that sett use is determined by a combination of drivers linked to diverse ecological mechanisms.

Setts surrounded by more diverse vegetation showed a higher frequency of use. This diversity of vegetation can be a consequence and not a driver of higher sett use by badgers. When involved in sett maintenance activities (e.g., excavation of new galleries or cleaning older ones) badgers alter the nutrient composition of the soil around the setts by surfacing material from deeper soil layers [51]. These nutrients then become available to plants, which could promote vegetation diversity. On the other hand, Mediterranean badgers consume shrubs and trees’ fleshy-fruits [52], and deposit their seeds in latrines outside but close-by the sett entrances, which may promote vegetation diversity [51].

Soil composition also appears to be highly influential. As reported for other badger populations, sandy soils seem to facilitate sett excavation and maintenance (e.g., [26,53]). Sandy soils are also well-drained, which may allow suitable internal conditions that are particularly important in Mediterranean areas were rain is highly concentrated in winter, often assuming a torrential regime [24].

Disturbance emerged as another important driver of sett use, although with an opposite than expected trend. While in a previous study [22] sett use was constrained by grazing pressure and understory clearing activities, here we detected a positive effect of grazing intensity occurring in sett vicinity during the study period. We believe this unexpected pattern may be linked to resource availability that might overrule grazing disturbance. With an increase of cattle presence there is an accumulation of bovine faeces in the field, which may promote coprophilous beetles’ abundance, which are one of badger’s main food resources in the region [54]. Although cattle may be present within the plot where the sett was located (averaging 45 ± 100 cows per hectare), cows may not equally use all area within the plot and not specifically the sett area which is dominated by shrubs. Thus, cattle may be promoting beetles abundance without adding an extra direct impact on the sett (which was corroborated by the lack of correlation between cattle pressure in the plots and the number of videos with cattle).

Other variables deemed to be important drivers of sett use in other regions (proximity to agriculture fields, houses and roads, understory clearcutting, forest cover or proximity to water sources) [22,25,26,55], were not considered as influential in this study. Agriculture fields clustered in the extreme north of the study area, and therefore had a negligible influence; also, no clearcutting activities occurred within the limits of the monitored setts (as the access of heavy machinery was prevented). As for the potential effect of disturbance factors, such as roads or residential areas, the studied farmstead is an agro-silvo-pastoral private area without any paved road and where traffic was negligible, being limited to a few four-wheel drive (4WD) vehicles and some agro-forestry machinery, only active during daytime. Moreover, inhabited houses are only located at the farmstead border limits. These specific characteristics of the study area seem to downscale the known effect of roads and housing on sett use patterns. The negligible effect of forest cover may be linked to the fact that most of the study area (c.a. 90%) is forested and all setts are located within forests. Although areas nearby streams may provide food resources to badgers [22], flooding events may lead badgers to avoid them to build setts [25].

### 4.2. Badger Social Group Structure and Density

Badger’ social groups were composed of 2–4 adult animals, which is a similar pattern to what has been described for other areas were badgers live in low densities, such as southern Spain (3.22 ± 0.32) [5], Bialowieza Primeval Forest, Poland (2.4 ± 0.6) [56], south-west (SW)Portugal (3–4) [12] and northern Italy (2.75) [57]. Groups are probably composed by the breeding pair and the sub-adults from the previous year [5]. However, in one occasion we detected in the same sett two sets of cubs of different ages, suggesting that sometimes young females can also reproduce. Small group size is probably linked to the lower availability and higher seasonality of food resources [56] of the Mediterranean environments, when compared to North-western Europe [5].

Estimated density (0.49–0.73 badgers/km^2^) corroborates our sixth hypothesis, i.e., low density scenario. This estimate is slightly higher than that recorded in Bialowieza Forest, Poland (0.21 badgers/km^2^) [56] or in Northern Moravia, Czech Republic (0.12) [7]. In the Mediterranean context, the estimation is similar to that mentioned for southern Spain (0.67 badgers/km^2^) [11], but much lower than for Italy (0.93–1.4 badgers/km^2^) [58], and higher than that reported for a southern Portuguese population (0.36–0.38 badgers/km^2^) inhabiting also a cork oak landscape [12].

Badger density is often linked to two main factors: food availability, and its seasonal variation [56], and/or availability of suitable sett-sites [13]. The somehow intermediate situation detected in our study area, given the European trends, may be linked to the agro-silvo-pastoral character of the region. Low food availability typical of Mediterranean environments, which is linked to high seasonal variation in resource availability and summer dryness stress (i.e., “Mediterraneity”) [59], might be compensated by the existence of human-linked food resources that badgers may use, such as game species feeding structures or the surplus of invertebrates linked to cattle presence [22], which may be available all year. This extra food availability may have increased the area’s carrying capacity for badgers, allowing density to increase to a threshold. The pedological characteristics of our study area seem more suitable than that found in the other studied region of southern Portugal [13], which may allow a higher availability of suitable sett sites.

However, we have to point out that the size of social groups and the density estimates may have been underestimated, as it was based on the simultaneous presence of animals outside the sett and at the focal area of the camera-traps. As no agonistic interaction was ever recorded among simultaneously observed individuals (which are more common among members of different groups [4]), the probability of including transient individuals belonging to other social groups can be considered negligible.

### 4.3. Breeding Phenology

The majority of copulatory behaviors and all long-duration mating events occurred in two distinct months (November and January). These types of events are often associated to an increase of fertilization probability [34]. Usually, fertilization occurs in the months following cub’s birth [4,34,60]. Thus, long-duration events in November may have involved young, non-pregnant females [60]. Such mating events may lead to immediate pregnancies that can result in cubs being born in mid to late January and emerge in late March or early April.

Cubs emergence from setts in March-April, corroborates the seventh hypothesis. Considering that usually the first emergence of cubs outside the sett occurs eight weeks after birth [4], cubs were born between early January and early February, as observed for other Mediterranean populations (i.e., southern Spain) [11]. Therefore, births in southern Europe occur earlier than in central and northern Europe, i.e., mid-January-March (peaking in February) in southern England, early March in Scotland and Sweden, or late March-April in Russia [4,11]. Usually, birth occur later in the northern part of badger range to match with the period of highest food availability, because females tend to adjust the higher energy demanding period (e.g., cub nursing) to higher resource availability and lower climatic challenges [61].

Litter size was consistent with those recorded in t many regions of the badger range [4], validating the eighth hypothesis. The estimated average litter per year per female was 2.7 cubs for the UK [58], 2.52 for Switzerland [62] and 2–3 for Southern Spain [11], values that do not differ much from the one we estimated.

## 5. Conclusions

While badger sett use patterns, social structure, density and breeding phenology are well known in the British islands and in some regions of central-east Europe (e.g., Poland) [4], data for Mediterranean populations are still scarce. Data shown in this study follows a general pattern regarding social group size and indicate an intermediate population density. This evidence, together with the breeding and cub emergence periods, indicates an ecological adaptation of this mustelid to local conditions, where human-related resources and mild environmental conditions allow badgers to reach higher densities than in many southern populations, and to reproduce earlier than their northern counterparts. This knowledge is crucial to properly address conservation challenges, and assign the adequate national conservation status for badgers, now that the Portuguese Red List data is under re-evaluation.

## Figures and Tables

**Table 1:** Hypotheses tested targeting three life-history patterns: sett use, density and breeding phenology. For each, we detail the underlying reasoning and the supporting references.

Pattern	Hypothesis	Reasoning	Supporting References
Density	**H1**—*Badger’s density will be lower when compared to the species core range area, but within the limits reported for Iberian populations*	Studies implemented in Iberia estimate a population density of 0.13–0.67 ind./km^2^, significantly lower than that estimated for central/west European populations (mean = 9.2 ind./km^2^, SD = 10.5)	[4,9,10,11,12]
Sett use pattern	**H2**—*Sett use is promoted by the occurrence of dense vegetation surrounding the sett*	In human-shaped landscapes, the occurrence of dense vegetation provides a more protective context, where animals may socially interact more safely (including cubs)	[24,25]
	**H3**—*Setts located in areas with a easily diggable and well-drained soil will be more frequently used*	Badgers prefer well drained and cohesive soils as sett sites	[26,27]
	**H4**—*Sett use is promoted by the proximity to feeding patches (e.g., olive yards, wildlife feeding stations)*	Sett location near patches providing food resources allows badgers to save energy and time in their foraging bouts	[22,28]
	**H5**—*Setts subject to high disturbance (e.g., cattle, roads proximity) will be less used by badgers*	Anthropogenic disturbance is known to affect sett use by badgers	[22,29,30,31]
	**H6**—*Setts located in areas where perceived competition can be high will be less used*	Evidence exist that the presence of wild boars constrains badgers presence, due to resource competition. Inter-group competition is also considered a passive range exclusion mechanism.	[32,33]
Reproduction phenology	**H7**—*Badger mating peaks in January/February and cubs will start emerging from the setts in April*	Badger mating can occur all year round, with two peaks in Winter/Spring (main) and Summer/Autumn; cubs emerge eight weeks after.	[11,34]
	**H8**—*Litters will be composed of 2–3 cubs*	Although available data for Portugal indicate 3-4 cubs/litter, the average values for most badger populations is lower	[12,34]

**Table 2:** Variables used to characterize each sett during the monthly monitoring visit, with reference to the hypothesis to which they were linked, the type of variable and its range/units.

	Variable	Description	Type	Range/Units
H2	Tree	Tree cover	Ordinal	1–5 (1—low; 5—high) ^1,2^
	Shrubs	Shrub cover	Continuous	Percentage ^1,2^
	Herbs	Herbaceous cover	Continuous	Percentage ^1,2^
	Shrub_H	Shrub mean high	Continuous	Cm ^1,2^
	Bare_soil	Bare soil cover	Continuous	Percentage ^1,2^
	Veg_div	Vegetation diversity	Continuous	0–1 (Simpson diversity index)
	Understory	Dominant understory	Categorical	4 Categories (Cattle pastures, Shrubland, Natural pastures, Natural pastures with shrubs)
	Land use	Type of land use	Categorical	4 categories (Cork oak woodland, Mixes wood, Natural pasture, Pine forest)
H3	Soil_mat	Main soil material	Categorical	4 categories (Sand, Rock, Silt/Clay, Roads ^3^) ^1^
	Soil	Type of soil		2 Categories (Podzols, Regosols)
H4	Dist_ol	Distance to the nearest olive yard	Continuous	m
	Dist_piv	Distance to the nearest watering pivot	Continuous	m
	Dist_feed	Distance to the nearest game artificial feeders	Continuous	m
	Dist_wat	Distance to the nearest water source	Continuous	m
H5	Grazz_16–17	Grazing pressure between 2016–2017	Continuous	Grazing intensity ^4^
	Grazz_07–17	Cumulative grazing pressure between 2007–2017	Continuous	Grazing intensity ^4^
	N_grazz	Number of consecutive years, since 2007, without cattle grazing	Continuous	Number of years
	Dist_road	Distance to the nearest paved road	Continuous	m
	Dist_road2	Distance to the nearest dirt road	Continuous	m
H6	Dist_sett	Distance to the nearest badger sett	Continuous	m
	Wildboar	Wild boar abundance	Continuous	Number of wild boar signs [40]

^1^ Variables collected in situ, with the minimum convex polygon encompassing all the sett entrances. ^2^ Variables used in the Principal Components Analysis (PCA). ^3^ Setts located under dirt roads. ^4^ Expressed as the number of cattle per day and per hectare, in the pot where each sett was located [35].

**Table 3:** Correlation between the original variables and the PCA components used in the modeling procedure. Variable description is detailed in Table 2.

Original Variable	Component 1	Component 2
Tree	−0.431	-
Shrubs	−0.501	−0.398
Herbs	−0.507	−0.132
Shrub_H	−0.547	−0.271
Bare_soil	−0.114	0.865

**Table 4:** Best models for each tested hypothesis (ΔAICc < 2). For each model we include the degree of freedom (df), the Akaike Information Criterion, corrected for small samples (AICc), the AICc difference for the lowest AICc in each hypothesis (ΔAICc), the Akaike weight (Weight), and the AICc difference for the lowest AICc of all produced models (Overall ΔAICc). Variables’ acronyms are described in Table 2.

	Model	df	LogLik	AICc	ΔAICc	Weight	Overall ΔAICc
	Null	1	−87.721	169.6	0	-	11.0
H2—Vegetation	Understory + Veg_div + PCA1	6	−73.950	163.0	0	0.209	4.4
	Understory + Veg_div	5	−75.574	163.3	0.3	0.182	4.7
	Veg_div	2	−70.981	164.3	1.3	0.107	5.7
	Veg_div + PCA1	3	−78.977	164.8	1.8	0.088	6.2
H3—Soil	Soil_mat	4	−75.027	159.4	0	0.781	0.8
H4—Food	Dist_ol	2	−82.114	168.6	0	0.346	10.0
	Dist_ol + Dist_wat	3	−81.902	170.6	2.0	0.128	12.0
H5—Disturbance	Dist_road2 + Grazz_16–17	3	−78.930	164.7	0	0.255	6.1
	Dist_road2 + Grazz_16–17 + N_grazz	4	−78.370	166.1	1.4	0.123	7.5
	Grazz_16–17	2	−81.036	166.5	1.8	0.104	7.9
H6—Competition	Dist_sett	2	−83.464	171.3	0	0.223	12.7
	Wildboar	2	−83.715	171.8	0.5	0.174	13.2
Combined hypothesis	Soil_mat + Veg_div	5	−73.204	158.6	0	0.151	0
	Dist_road2 + Grazz_16-17 + Veg_div	4	−74.792	159.0	0.4	0.123	0.4
	Soil_mat + Grazz_16-17 + Veg_div	6	−72.095	159.3	0.7	0.104	0.7
	Soil_mat	4	−75.027	159.4	0.8	0.097	0.8
	Veg_div + Grazz_16-17	3	−76.340	159.5	0.9	0.095	0.9

**Table 5:** Variables included in the average model of the best models produced to explain the variation in sett use frequency (Combined hypothesis—Table 4). For each variable the coefficient (β), standard error (SE), z-value and significance (*p*), 95% confidence intervals (95% CI) and relative importance are presented. (See Table 2 for variables’ description).

Variable	β	SE	*z*-Value	*p*	95% CI	Relative Importance
Intercept	0.719	0.601	1.175	0.240	[−0.480, 1.919]	-
Soil_mat (Silt/clay)	−0.740	0.244	2.919	0.004	[−1.237, −0.243]	0.62
Soil_mat (Rock)	−0.115	0.246	0.450	0.653	[−0.618, 0.388]	0.62
Soil_mat (Roads)	0.264	0.403	0.629	0.530	[−0.559, 1.087]	0.62
Veg_div	1.989	0.862	2.122	0.034	[0.145, 3.651]	0.83
Dist_road2	−0.157	0.091	1.662	0.096	[−0.342, 0.028]	0.22
Grazz_16-17	0.194	0.092	2.041	0.041	[0.007, 0.381]	0.56

## Data Availability

The datasets generated during the current study are available from the corresponding author on reasonable.

## Supplementary Materials

The following are available online at www.mdpi.com/article/10.3390/ani11092663/s1, Figure S1: Badger main sett showing fresh nest material at one of the active entrances, Figure S2: Installation of a camera-traps used to monitor one of badger’s main setts within Charneca do Infantado farmstead, Central West Portugal, Figure S3: European badger (*Meles meles*) cub detected during our field work outside one of the main setts in March 2017.
