# Supplementary material for: Sett Use, Density and Breeding Phenology of Badgers in Mediterranean Agro-Sylvo-Pastoral Systems
Source: Animals (Basel). 2021 Sep 10;11(9):2663. doi: 10.3390/ani11092663 (PMC8471938; doi:10.3390/ani11092663)
Supplement: Supplementary file 1 [file animals-11-02663-s001.zip › animals-1345164-supplementary.pdf]

Supplementary Materials

Sett Use, Density and Breeding Phenology of Badgers in Mediterranean Agro-Sylvo-Pastoral Systems

Marcelo Silva [1,†], Luís Miguel Rosalino [1,†], Sandra Alcobia [1] and Margarida Santos-Reis [1,*]

[1] cE3c - Centre for Ecology, Evolution and Environmental Changes, Faculdade de Ciências, Universidade de Lisboa, 1749-016, Lisboa, Portugal; Marcelo_Gomes_Silva12@hotmail.com (M.S.); lmrosalino@fc.ul.pt (L.M.R.), alcobiasandra@gmail.com (S.A.)

† These authors contributed equally to this work

* Correspondence: Correspondence: mmreis@fc.ul.pt

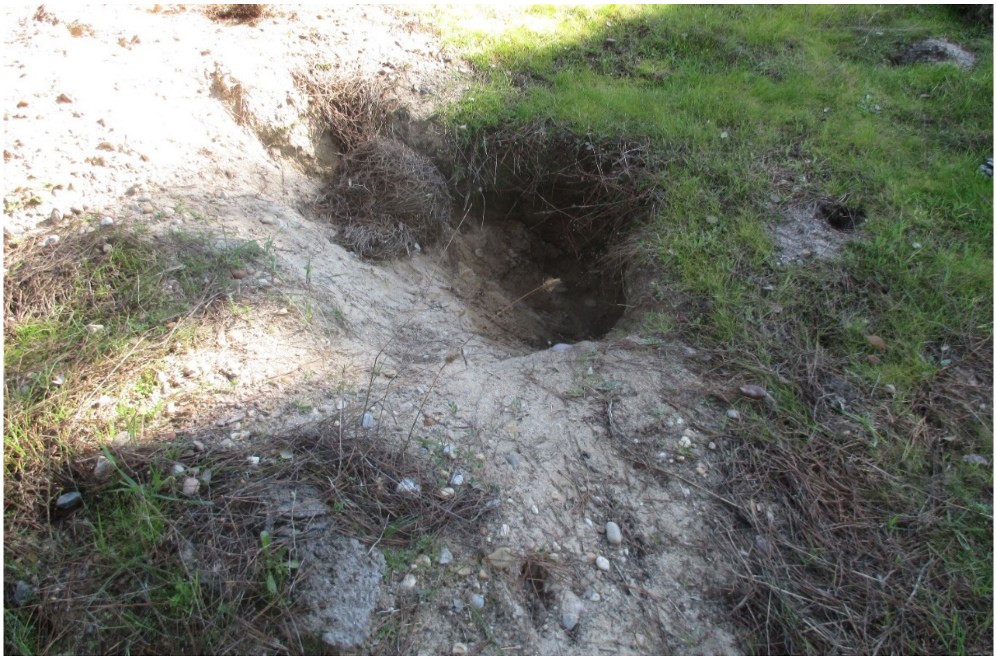

Figure S1. Badger main sett showing fresh nest material at one of the active entrances.

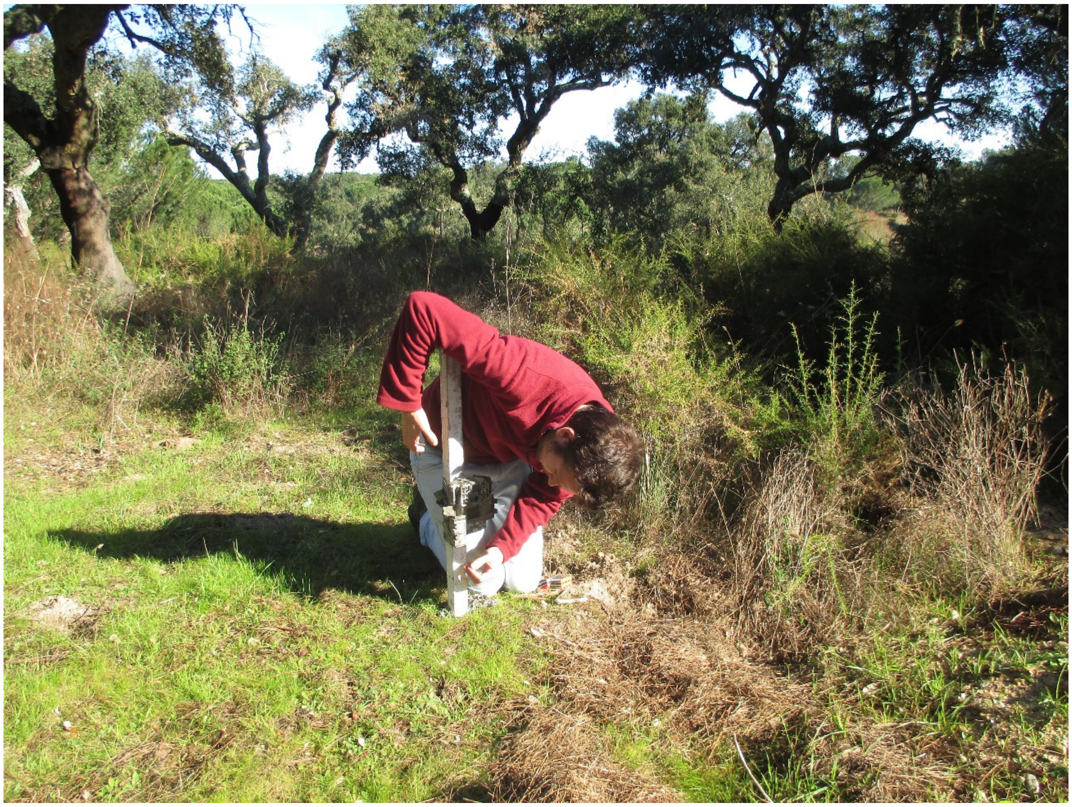

Figure S2. Installation of a camera-traps used to monitor one of badger's main setts within Charneca do Infantado farmstead, Central West Portugal.

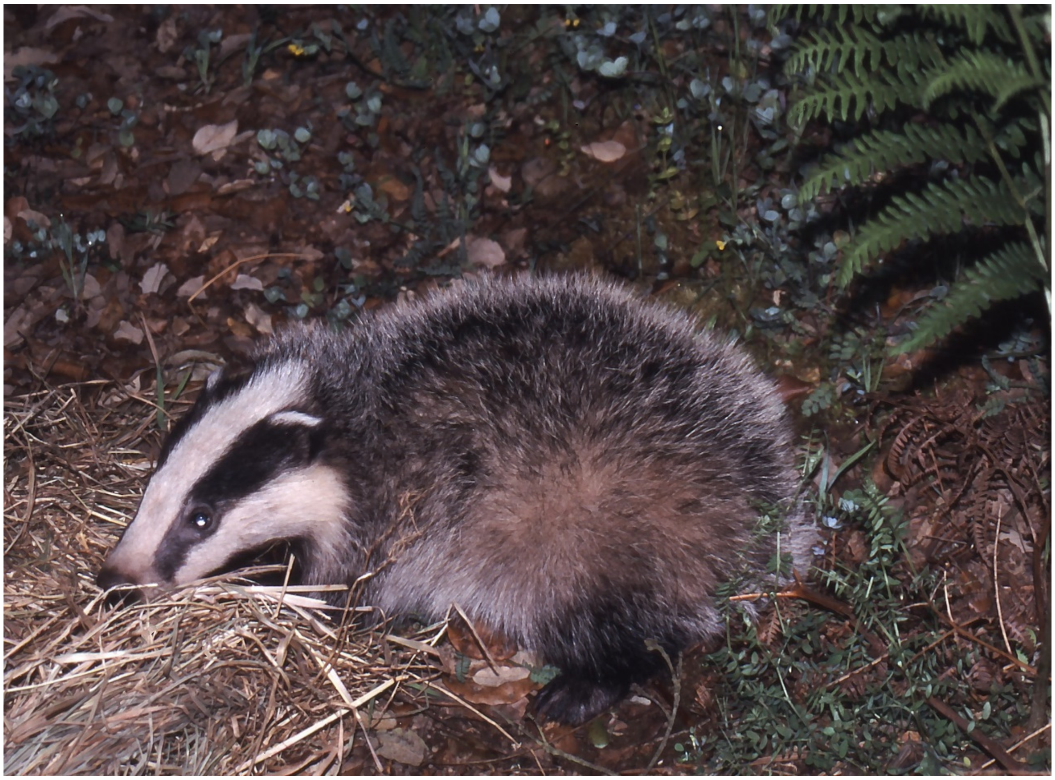

Figure S3. European badger (*Meles meles*) cub detected during our field work outside one of the main setts in March 2017.
